# Th17 Cells and the IL-23/IL-17 Axis in the Pathogenesis of Periodontitis and Immune-Mediated Inflammatory Diseases

**DOI:** 10.3390/ijms20143394

**Published:** 2019-07-10

**Authors:** Kübra Bunte, Thomas Beikler

**Affiliations:** Department of Periodontics, Preventive and Restorative Dentistry, University Medical Centre Hamburg-Eppendorf, 20246 Hamburg, Germany

**Keywords:** periodontitis, psoriasis, rheumatoid arthritis, Crohn’s disease, ulcerative colitis, systemic lupus erythematosus, Sjögren syndrome, type 1 diabetes mellitus, cytokines, Th17 cells, interleukin-17, interleukin-23

## Abstract

Innate immunity represents the semi-specific first line of defense and provides the initial host response to tissue injury, trauma, and pathogens. Innate immunity activates the adaptive immunity, and both act highly regulated together to establish and maintain tissue homeostasis. Any dysregulation of this interaction can result in chronic inflammation and autoimmunity and is thought to be a major underlying cause in the initiation and progression of highly prevalent immune-mediated inflammatory diseases (IMIDs) such as psoriasis, rheumatoid arthritis, inflammatory bowel diseases among others, and periodontitis. Th1 and Th2 cells of the adaptive immune system are the major players in the pathogenesis of IMIDs. In addition, Th17 cells, their key cytokine IL-17, and IL-23 seem to play pivotal roles. This review aims to provide an overview of the current knowledge about the differentiation of Th17 cells and the role of the IL-17/IL-23 axis in the pathogenesis of IMIDs. Moreover, it aims to review the association of these IMIDs with periodontitis and briefly discusses the therapeutic potential of agents that modulate the IL-17/IL-23 axis.

## 1. Introduction

Innate immunity represents the semi-specific first line of defense for all primitive and complex multicellular organisms and provides the initial acute inflammatory reaction to tissue injury, trauma, or pathogens [1]. Innate immunity is a rather unspecific and immediate reaction that recruits immune cells to the injury or infection site through various cytokines (e.g., prostaglandins, tumor necrosis factor (TNF), interleukin (IL)-1β, and others). Moreover, it promotes phagocytosis, and activates the complement and adaptive immune system [2]. In contrast to the innate immune system, the activation of the adaptive immune system results in an antigen specific host response that is mediated by T and B cells. It usually requires more time than the innate immune system to react since it turns progenitor cells into regulatory and effector cells with distinct functions, such as (i) self and non-self-antigen recognition, (ii) enhanced elimination of pathogens or infected cells, and (iii) development of immune memory [3]. 

The innate and adaptive immunity interaction represents a complex system. They are equally required to establish and maintain health and tissue homeostasis and both comprise cellular and humoral immunity components. Humoral immunity refers to antigen-specific antibody production to neutralize toxins and pathogens, mediation of allergic reactions and autoimmunity, generation of immune memory cells, and stimulation of cytokine secretion; whereas, cellular immunity eradicates pathogens by macrophages or natural killer cells, eliminates intracellular bacteria via induction of cytotoxic T-cell-mediated apoptosis, and stimulates tissue cells, such as fibroblasts, to secrete cytokines that further modulate innate and adaptive immune system responses [4]. The inflammation triggered by pathogens, tissue injury, or trauma is typically self-limiting and results in tissue repair and reestablishment of tissue homeostasis following elimination of the cause. However, an alteration of the regular immune response may result in persistence of the acute inflammation, its transition into chronic inflammation, and could even induce autoimmune reactions in susceptible individuals. This altered inflammatory response is believed to be a major underlying cause in the initiation and progression of disorders, such as immune-mediated inflammatory diseases (IMIDs) [5]. 

IMIDs are the definition of a group of seemingly unrelated diseases that share common inflammatory pathways and are triggered by or result in the dysregulation of innate and adaptive immune system functions [6]. The definitive etiology is still unclear; however, genetic susceptibility and environmental factors such as infection and trauma may initiate these conditions, that include, but are not limited to, psoriasis, rheumatoid arthritis, inflammatory bowel diseases, systemic lupus erythematosus, Sjögren syndrome, and type 1 diabetes [7,8]. Any organ system may be inflicted, and individuals may encounter a considerable reduction in quality of life, significant morbidity, and reduced lifespan [6]. 

Initiated and perpetuated by a dysbiotic oral microbiome, periodontitis is the most common inflammatory disease of tooth supporting tissues, with a high prevalence of up to 70% among the world’s dentate adults [9,10]. The host immune and inflammatory responses disrupted by a dysbiotic microbiome are considered to be the main cause for the initiation, establishment, and progression of periodontal inflammation and tissue breakdown [11]. If left untreated, periodontitis results in progressive periodontal attachment and alveolar bone loss, which subsequently results in tooth loss. Co-existence of an IMID with another systemic inflammatory/autoimmune disease or periodontitis is not uncommon, e.g., 20–30% of patients with psoriasis will eventually develop psoriatic arthritis (PsA) [12]. Although the pathogenesis of IMIDs is not yet entirely understood and elucidated and a causative bidirectional relationship between IMIDs and periodontitis has not been proven yet, the comorbidities indicate the involvement of a dysbalanced inflammatory cytokine network in the disease processes [13,14,15]. 

As mentioned before, cytokines and antigen presentation attract and activate adaptive immune system cells. In this regard, CD4^+^ helper T cells are pivotal players [16]. CD4^+^ helper T cells differentiate into regulatory and effector T cell subsets i.e., Th1, Th2, Th17, follicular helper T (Tfh) cells, and regulatory T cells (Tregs), following activation. Until the discovery of other cell lineages, Th1 and Th2 were thought to be the only cells differentiating from progenitor CD4^+^ helper T cells [17]. In this classical Th1/Th2 paradigm, Th1 cells mainly produce interleukin(IL)-2 and interferon gamma (IFN-γ) and are involved in cellular immunity [18]. Th2 cells are mainly responsible for humoral immunity via the activation of B cells, mast cells, and production of immunoglobulin E, and primarily produce IL-4, IL-5, and IL-13 [19]. The first findings that indicated the existence of a novel effector population of CD4^+^ helper T cells were provided by an animal model of multiple sclerosis (experimental autoimmune encephalitis, EAE) [20]. According to the Th1/Th2 paradigm, it was initially hypothesized that IL-12 and, hence, Th1 cells and IFNγ were playing the central role in this inflammatory disease; however, it was demonstrated that functional Th1 pathways downregulate the onset and progression of EAE in IFNγ- and IL-12-deficient mice [21,22,23]. Later it was demonstrated that indeed IL-23, and not IL-12, was involved in the EAE pathogenesis [24]. This was further substantiated when the transfer of IL-17 producing T cells to healthy mice was sufficient to induce EAE [25]. In addition, IL-23-deficient animals were shown to be unable to recruit IL-17 producing T cells [26]. These and other studies led to the identification of a novel CD4^+^ helper T cell subset named T helper 17 (Th17) cell lineage that was characterized by IL-17 production, a cytokine that Th1 and Th2 are not able to produce [17,27]. 

The Th1/Th2 paradigm provided a framework for understanding the pathogenesis of several conditions, such as psoriasis, rheumatoid arthritis, inflammatory bowel diseases, and periodontitis. However, the identification of Th17 cells as a distinct lineage of CD4^+^ helper T cells has greatly expanded the understanding of autoimmunity and inflammation and filled in some of the missing gaps in host immunity that could not be fully explained by the Th1/Th2. Although there is still much to be elucidated, this review aims to provide an overview of the current knowledge about the Th17 cell lineage, the role of its key cytokine IL-17, the IL-17/IL-23 axis in health and disease, and strategies that target IL-17 related pathways. Understanding the similar mechanisms that drive the pathogenesis of these diseases will support an interdisciplinary approach between medical doctors and dentists, and thus allow proper screening, prevention, and early treatment. 

## 2. Differentiation and Regulation of Th17 Cells

**Figure 1:**
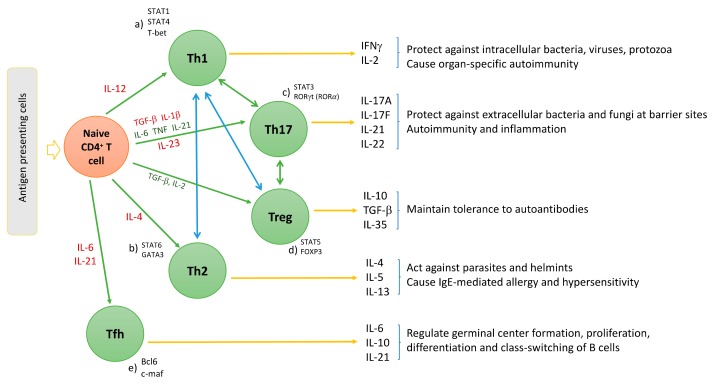
Differentiation of naïve CD4^+^ cells into Th1, Th2, Th17, regulatory T cells (Treg), and follicular helper T (Tfh) cells. IL-12 leads to Th1-, IL-4 leads to Th2-, TGFβ leads to Treg-differentiation. Th17 cells differentiation is initiated by IL-1β, TGFβ, and further stimulated by TNF, IL-6, and IL-21; Th17 cells are maintained and expanded by IL-23. The transcription factors that governs Th cell subset differentiation are different, (**a**) STAT1, STAT4, and T-bet drive Th1; (**b**) STAT6 and GATA3 drive Th2; (**c**) STAT3 and RORγt (RORα, in the absence of RORγt) manage Th17 cell; (**d**) FOXP3 and STAT5 govern Treg cell differentiation; and (**e**) Bcl6 and c-maf are the regulators of Tfh cells differentiation.

The polarization of progenitor CD4^+^ helper T cells involves specific signal transduction mechanisms, distinct transcription factors, and local cytokine profiles for each T helper cell lineage (Figure 1). For instance, the differentiation of IFNγ producing Th1 cells is induced by IL-12 (a heterodimer consisting of a p35 and a p40 subunit) and by activation of the transcription factor STAT4 (Figure 1d) [28]. Also, IFNγ stimulates STAT1 and T-bet (a downstream transcription factor) expression in CD4^+^ helper T cells, which in turn upregulates Th1 specific gene expression (Figure 1a) [28]. Th2 cell differentiation is, however, mainly driven by IL-4. IL-4 increases STAT6 and GATA-3 expression; thus, upregulates of Th2 differentiation (Figure 1b) [29]. The differentiation of Th1 and Th2 is mutually antagonistic, primarily due to the antagonism of IFNγ and IL-4 on molecular and cellular levels [30]. 

**Figure 2:**
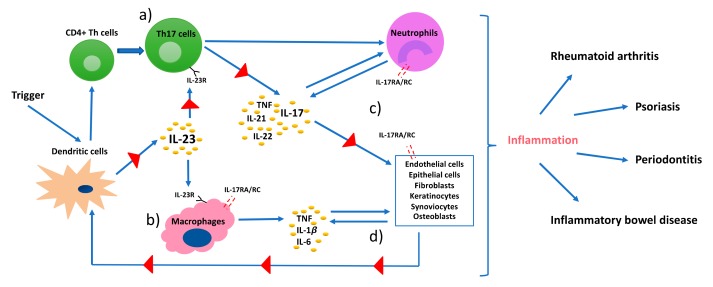
A pathophysiology model of immune-mediated inflammatory diseases. Dendritic cells (DCs) are triggered by a stimulus, such as environmental stress or infection, present the antigen that leads to differentiation of CD4^+^ helper T cells, and release IL-23. IL-23 stimulates the production of proinflammatory cytokines, such as TNF, IL-1β, and IL-17 from (**a**) Th17 cells, and IL-6 from (**b**) macrophages and DCs. (**c**) IL-17 interacts with IL-17RA/RC complex on receptor carrying cells. These cells further produce inflammatory mediators that regulate functionality of DCs and create a self-sustaining feedback loop via IL-23 (**d**).

Distinct from the differentiation of Th1 and Th2 cell lineages, Th17 cell differentiation is induced by STAT3 and retinoid acid related-orphan nuclear receptor γt (RORγt) that work synergistically with one another (Figure 1c) [31]. Similar to Th1/Th2 cell interaction, Tregs and Th17 cells are also maintained in an equilibrium. The transcription factor forkhead box P3 (FOXP3) is the negative regulator of RORγt and maintains the tolerance of the organism to self-antigens by inducing the differentiation of Tregs via STAT6 and downregulating differentiation of Th17 cells (Figure 1d) [32]. However, the Treg/Th17 balance is shifted in favor of Th17 in the presence of proinflammatory cytokines. In this regard, transforming growth factor beta (TGFβ) independently initiates the differentiation of Tregs from naïve CD4^+^ helper T cells by activating FOXP3; whereas, it induces the differentiation of Th17 cells in the presence of IL-6 in mice and IL-1β in humans (Figure 1c,d) [33,34]. Exposure to TGFβ/IL-1β and IL-6 results in inhibition of FOXP3 and activation of RORγt, thus initiating the differentiation cascade of Th17 cells [35]. Furthermore, TNF and IL-1β can synergistically increase IL-6 production and contribute to further Th17 cell differentiation [36]. Following activation, RORγt promotes the expression of IL-17 and IL-23 receptor (IL-23R) [37]. In the absence of RORγt, RORα has been shown to stimulate IL-17 expression [38]. IL-23 is produced by antigen-presenting cells, such as dendritic cells and monocytes/macrophages upon activation and initiates signaling by binding to IL-23R, which in return increases the expression of RORγt and IL-17 via STAT3 (Figure 1c and Figure 2) [39]. However, IL-23 is not able to induce Th17 cell development from naïve CD4+ helper T cells alone by itself, because IL-23R is expressed only after the differentiation of a naïve CD4^+^ helper T cell into a Th17 cell is initiated [17]. This suggests that the role of IL-23 is rather to be seen in the maintenance, expansion, and survival of Th17 cells than in the initiation of their differentiation. IL-23 is able to maintain and expand Th17 cell populations through a positive feedback loop that upregulates IL-17, RORγt, along with TNF, IL-1β, and IL-6 [40]. In addition, IL-21 was also demonstrated to amplify Th17 cell differentiation in cooperation with TGFβ in an IL-6-independent manner through its feedback function on developing Th17 cells [31,41]. 

Some other general transcription factors, such as basic leucine zipper transcription factor ATF-like (BATF) and interferon regulatory factor 4 (IRF4), are also involved in the differentiation of naïve CD4+ helper T cells [42,43]. As such, IRF4 deficient mice were shown to have decreased levels of RORγt and increased levels of FOXP3 [44]. These non-nuclear transcription factors are, however, broadly expressed in both health and disease. Therefore, STAT3-induced RORγt is suggested to be the key transcriptional factor involved in Th17 differentiation. This was also supported by the finding that RORγt (*Rorc^-/-^*) deficient CD4+ helper T cells failed to differentiate into Th17 [45]. 

Th17 cells are typically involved in vigorous proinflammatory responses, yet they also remain in the tissues, such as skin and mucosas as quiescent cells [46]. The inflammatory functions of Th17 cells depends on the different combinations of expressed cytokines in the local environment. For instance, Th17 cells were demonstrated to produce the anti-inflammatory cytokine IL-10 when stimulated with IFNα/β, thus downregulating their pathogenic functions [47]. In contrast to that, IL-23 was shown to reduce the expression levels of IL-10 in developing Th17 cells and induce pro-inflammatory Th17 cells that produce IL-17 [48]. Moreover, different subsets of TGF have also been demonstrated to induce distinct functions in Th17 cells. For instance, TGFβ1-and-IL-6-induced Th17 cells were unable to cause EAE in the absence of IL-23, whereas TGFβ3-induced Th17 cells presented highly pathogenic functions [49]. Moreover, Th-cells, especially Th17 cells, exhibit high phenotypic and functional plasticity, which means that they can transdifferentiate into other T cell subsets in different inflammatory settings [50]. As mentioned, the involvement of IL-6 during early stages of TGFβ-induced Treg differentiation can convert Treg cells into pathogenic Th17 cells [51]. Mature Th17 cells can furthermore be transformed by IL-6 into IFNγ producing Th1 cells [52]. In conclusion, differentiation and regulation of Th17 cells is mediated by a complex cytokine and transcription factor network, which may result in both pathologic and non-pathologic effector functions in inflammatory and autoimmune diseases.

## 3. Th17/IL-17 in Immunoprotection and Immunopathology

IL-17 is the key cytokine produced by Th17 cells and exerts versatile functions. The IL-17 receptor (IL-17R) lacks a homologous structure to other proteins; therefore, IL-17 cytokines are classified as a distinct family [53]. The IL-17 family consists of six ligands, IL-17A to IL-17F, that share similar structures and can exist as homodimers or form heterodimers, such as IL17A/F. IL-17A and IL-17F (henceforth referred to as IL-17) are produced by Th17 cells. They present 50% of structural homology and their intracellular signal transduction is dependent on the presence of the receptor heterodimer complex formed by the subunits IL-17 receptor A (IL-17RA) and IL-17 receptor C (IL-17RC) [54]. IL-17 receptor is expressed on a broad range of cells, including osteoblasts, endothelial cells, epithelial cells, fibroblast-like synoviocytes, chrondrocytes, fibroblasts, keratinocytes, and macrophages (Figure 2) [40,55,56,57]. Although IL-17 is predominantly produced by Th17 cells, it can also be expressed by natural killer T (NKT) cells, γδ T cells, lymphoid tissue inducer cells, neutrophils, and group 3 innate lymphoid cells (ILC3s) (Figure 2) [58,59]. Moreover, Th17 cells not only produce IL-17, but also IL-21, IL-22, IFNγ, and TNF and can express CCR6, i.e., the receptor of chemokine (C-C motif) ligand 20 (CCL20) that directs IL-17 producing cells to the epithelial barrier sites [60]. IL-17-producing cells accumulate at mucosal surfaces of the oral cavity, gastrointestinal tract, lungs, vagina, and skin epithelium, and regulate protective immunity against extracellular pathogens (especially Gram-negative bacteria and fungi) by maintaining barrier integrity, promoting antimicrobial factors, and activating granulopoiesis [31,59]. Furthermore, the Th17-cytokine IL-22 was shown to contribute to IL-17-induced protective functions by enhancing the production of antimicrobial peptides and recruiting neutrophils [61]. Therefore, the disruption of IL-17 production or signaling can increase the susceptibility to bacterial and fungal infections. In this context, IL-17 receptor deficiency is associated with mortality in mice due to the inability to recruit neutrophils when being infected with *Klebsiella pneumoniae* [62]. Moreover, genetic defects in IL-17 immunity, such as in STAT3 (manifested as hyper-IgE syndrome), result in recurrent and persistent Candida spp. infections; e.g., chronic mucocutaneous candidiasis [63]. Direct IL-17 inhibition with monoclonal antibodies in patients with psoriasis or psoriatic arthritis has been shown to increase the risk of candida infections; similarly, the reactivation of latent tuberculosis infection was observed in patients treated with TNF-inhibitors [64,65]. Th17 cells are also regularly maintained in the gingival tissues, suggesting a protective role in the oral barrier; however, the mechanism that maintains these cells in the tissue is yet to be clarified [66]. Interestingly, IL-17R lacking mice are shown to be more susceptible to *Porphyromonas gingivalis (Pg)-*induced bone loss, suggesting a protective role of IL-17 in bone remodeling and homeostasis [67]. Although IL-17 may exert protective functions, several clinical human studies indicate that excessive production of IL-17 is associated with periodontitis, as well as psoriasis, rheumatoid arthritis, and other IMIDs [52,68]. 

IL-17 by itself is a weak inducer of inflammation; its potent inflammatory effects are derived from its synergistic functions with other cytokines and its ability to recruit and maintain inflammatory cells, such as neutrophils. Moreover, IL-17 eases the access of these cells to tissues through regulating the expression of chemokine (C-X-C motif) ligands (CXCL1, CXCL2, CXCL5, CXCL8), CCL20, IL-1β, MMPs, PGE2, and granulocyte- and granulocyte-macrophage colony-stimulating factors (G-CSF and GM-CSF) [51,59]. GM-CSF and G-CSF are the primary regulators of granulopoiesis and neutrophil release from the bone marrow. Its upregulation by IL-17 can lead to excessive activation and mobilization of neutrophils and production of chemokines that increase neutrophil diapedesis, which in return intensifies tissue damage. In addition to that, IL-17 works synergistically with other inflammatory cytokines, such as IL-1β, to increase CCL20 production from human gingival fibroblasts and stimulates further recruitment of Th17 cells; hence, IL-17 production [52]. Typically, neutrophils are found in inflamed periodontal tissues, but infiltration of activated Th17 cells can also be observed in inflamed sites [69,70]. This explains the upregulation of IL-17 in gingival crevicular fluid, bone, and gingiva of periodontitis patients [71,72,73]. In addition to the locally increased production of IL-17, the serum levels of IL-17 were also detected to be up to nine-fold higher in periodontitis patients compared to healthy controls [74]. Moreover, RORγt encoding gene (*Rorc*) expression levels were reported to be significantly higher in periodontal tissues of patients with periodontitis compared to healthy controls [75]. At last, the presence of T cell populations that can produce IL-17 in both, health and disease, demonstrates the versatile role of this cytokine.

### 3.1. IL-17 Dependent Processes in Psoriasis and Association with Periodontitis

Characterized by abnormal and rapid keratinocyte differentiation and thickened epidermis, psoriasis is an immune-mediated inflammatory skin disorder that affects 0.1–3% of the general population [76]. Although psoriasis is manifested in 90% of the cases in a skin plaque form called “psoriasis vulgaris”, it can also manifest itself in guttate, pustular, and erythrodermic subtypes, as well as in psoriatic arthritis [77,78]. Psoriasis is also associated with other systemic conditions such as hypertension, atherosclerosis, and diabetes [79,80]. The definitive etiology of psoriatic diseases is not clear yet; however, genetic, epigenetic, and environmental factors seem to contribute to the atypical activation of the innate and adaptive immune system resulting in disease onset, progression, and comorbidities [81,82]. Streptococcal peptidoglycan was also suggested to be involved in the pathogenesis of psoriasis; however, whether the skin microbiota is significant as a primary cause or a contributing factor remains to be elucidated [83]. Psoriasis has been primarily considered a Th1-mediated disease, today it is known that the IL-23/IL-17 axis governs the accelerated progress of inflammation [84,85].

**Figure 3:**
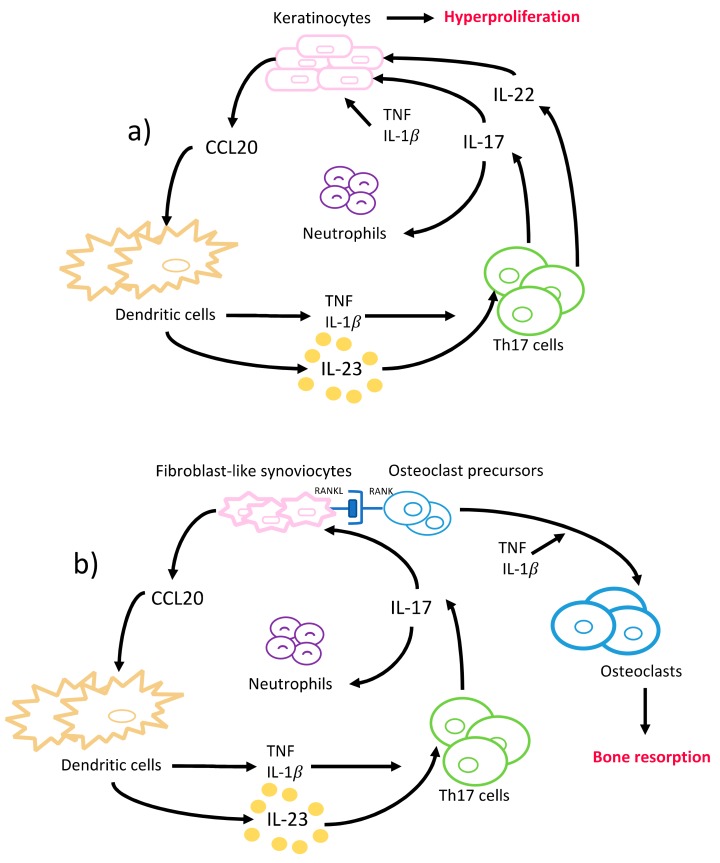
A simplified model of Th17 cells and the cytokines involved in the pathogenesis of (**a**) psoriasis and (**b**) rheumatoid arthritis. This figure can also be interpreted as a model of pathogenesis of gingival, periodontal, and oral mucosal inflammatory diseases, where cells such as gingival fibroblasts, keratinocytes, and epithelial cells are involved.

Briefly, IL-17 attracts neutrophils to the epidermis of psoriatic skin lesions through neutrophil chemoattractants and recruits additional dendritic cells via upregulation of CCL20 release from keratinocytes (Figure 3a). The dendritic cells release TNF and IL-1β and activate further differentiation of Th17 cells, and thus increased IL-17 production. IL-17 disrupts the integrity of the skin barrier through downregulation of filaggrin and adhesion molecule expression from keratinocytes, and further induces keratinocyte hyperproliferation (Figure 3a) [86]. The disease severity correlates with the numbers of IL-17 producing T cells in psoriatic lesions and their ability to increase keratinocyte proliferation and IL-17 production [87,88]. In addition, IL-22 aggravates psoriasis lesions in synergy with IL-17, although it exerts protective functions in non-psoriatic skin (Figure 3a) [89]. Also, the expression of RORγt, IL-1β, IL-6, and IL-23 was reported to be increased in psoriatic skin lesions compared to the non-psoriatic skin of patients and healthy volunteers; whereas the levels of anti-inflammatory cytokines, i.e., IL-4 and IL-10, were found to be decreased [14,86]. Besides the local changes TNF, IFNγ, IL-2, IL-17, and IL-22 levels were found to be increased in serum of psoriasis patients [14]. Increased levels of TNF, IL-1β, and IL-22 augment the inflammatory effects of IL-17 by enhancing the expression of TNF receptors, suggesting a synergistic interplay between these cytokines in psoriasis pathogenesis [90]. 

The role of a dysbiotic skin microbiota is controversially discussed in the pathogenesis of psoriasis. In periodontitis, however, a dysbiotic oral microbiome is known to contribute to the disease onset and progression [11]. Interestingly, it has recently been suggested that IL-17 creates a shift towards a highly pathologic bacterial environment, hence aggravating the periodontal inflammation like a vicious circle [91]. In addition, IL-23-dependent IL-17 production led to bacterial overgrowth, as demonstrated in leukocyte adhesion deficient (LAD) I periodontal phenotype mice and its inhibition reduced the bacterial overgrowth, which linked overexpression of IL-17 to microbial dysbiosis in periodontitis [92]. LAD1 is an immunodeficiency caused by a genetic mutation that results in defective neutrophil adhesion and tissue transmigration and is characterized by recurrent skin infections, oral ulcers, severe periodontal inflammation, and bone loss [93]. Although severe periodontitis in LAD1 was solely attributed to the lack of neutrophil surveillance in gingival and periodontal tissues, recent findings demonstrate that the excessive inflammatory response, mediated by IL-17, contributes to its manifestation [93,94].

Many retrospective and case-control studies have reported an association between psoriatic diseases, mainly psoriasis vulgaris and psoriatic arthritis, and periodontal disease [95,96,97]. A large 5-year follow-up cohort study indicated that in psoriasis patients (psoriasis subtypes not specified), chronic periodontitis showed an incidence rate of 1.88 per 1000 patient-years compared to 1.22 in the control group [98]. In accordance with these results, a longitudinal cohort study conducted in the Danish population retrospectively screened more than 5 million subjects to assess the risk for the development of periodontitis after the diagnosis of mild and severe psoriasis, and/or psoriatic arthritis [99]. Following adjustment for age, sex, co-morbidities, and smoking, the results demonstrated an increased risk for periodontitis among patients with psoriatic diseases, with the highest risk found in patients with psoriatic arthritis (risk ratio of 1.66, 2.24 and 3.48 for mild psoriasis, severe psoriasis and psoriatic arthritis, respectively). Moreover, the severity of periodontitis has been shown to be correlated with psoriasis severity [100]. Both diseases share common risk factors, i.e., smoking, obesity, and these risk factors can independently contribute to disease manifestation and severity. The risk for periodontitis was increased by six-fold in smoking psoriasis subjects compared to non-smoking psoriasis subjects [101]. Also, a significant correlation between periodontitis and psoriasis was reported after adjustment for smoking [102]. Although none of these studies could demonstrate a bidirectional causal relationship, regular periodontal screening seems to be reasonable in individuals with psoriatic diseases, since the risk for periodontitis is increased, especially in the presence of shared risk factors that negatively influence both diseases. 

### 3.2. IL-17 Dependent Processes in Rheumatoid Arthritis and Association with Periodontitis

Rheumatoid arthritis (RA) is a chronic inflammatory and autoimmune disease of the joints, characterized by the presence of rheumatic factor and anti-citrullinated protein antibodies (ACPAs) and persistent symmetrical and erosive polyarthritis, which results in progressive articular bone and cartilage destruction [103]. The prevalence is 0.5–1% among adults, and women are affected two to three times more frequently than men [55]. The development of rheumatoid arthritis is also considered to be of genetic, epigenetic, and environmental origin [104]. HLA-DR1 and HLA-DR4-positive individuals are reported to be at significantly higher risk for manifesting ACPA-positive RA, although only about 67% of the rheumatoid arthritis patients are ACPA-positive in the early stages [105,106]. Synovitis, the inflammation of the synovial membrane in joints, causes the clinical signs and symptoms of RA. Similar to psoriasis, an interactive and complex network of immune system cells and cytokines are involved in RA pathogenesis. ACPA establishes an abnormal immune response over time that promotes inflammation, and the synovial membrane becomes highly vascularized and infiltrated with fibroblasts, macrophages, T- and B- cells, plasma cells, mast cells, dendritic cells, and neutrophils [107]. Increased TNF in the synovial fluid induces IL-1β production, T- and B-cell activation, and a cascade of inflammatory reactions that is mainly led by dendritic- and Th17-cells, that eventually leads to articular bone and cartilage destruction (Figure 3b) [57]. In the RA-affected synovium, IL-17, IL-1β, and TNF act together to induce the chemotaxis of T cells and immature dendritic cells. This results in an upregulated CCL20 production in synoviocytes and an overall increased concentration of proinflammatory cytokines in the synovial tissues [108]. The role of IL-17 in rheumatoid arthritis was clearly demonstrated when long-term intra-articular administration of IL-17 in mice resulted in rheumatoid arthritis key features like inflammation, articular bone, and cartilage destruction [109]. Furthermore, several animal models of rheumatoid arthritis reported a reduced incidence, severity, and even resistance to disease upon the induction of IL-17 deficiency [110,111,112].

In addition to the inflammatory effects of IL-17, the osteoclastogenic character of IL-17 puts it in the focus of interest in bone-destructing diseases. The information regarding the effects of IL-17 on bone metabolism mainly originates from studies conducted on rheumatoid arthritis; however, several studies also report its effects on periodontal bone destruction. As mentioned before, dendritic cells that are present in the synovial fluid increase the release of TNF, IL-1β, IL-6, and IL-23 and stimulate Th17-cell differentiation, and thus IL-17 production in rheumatoid arthritis joints [55]. IL-17 disturbs the bone homeostasis by inducing osteoclastogenesis, which results in extensive and rapid bone destruction [113]. This is initiated by the increase of the receptor activator of nuclear factor kappa-B ligand (RANKL) expression on fibroblasts and osteoblasts by IL-17. RANKL interacts with the receptor activator of nuclear factor kappa-B (RANK) on dendritic cells and osteoclasts and activates synovial macrophages to secrete IL-1β and TNF—both are known to induce osteoclastogenesis [114]. Similarly to the mechanism in RA, IL-17 induces RANKL expression by stimulating MMP-1, MMP-3, IL-6, and IL-8 secretion from human gingival fibroblasts and TNF release from macrophages in periodontal tissues [115]. IL-17 levels are shown to be positively correlated to RANKL expression levels in periodontal ligament cells [116,117]. Furthermore, upregulated TNF and IL-17 can synergistically lead to further stimulation of the fibroblasts and epithelial cells to secrete IL-6, IL-8, PGE2, and the neutrophil chemoattractants; thus, intensifying the inflammation [35]. Independently from IL-17, IL-22 was also demonstrated to increase synovial inflammation in rheumatoid arthritis joints and clinical attachment loss in periodontitis patients, similarly to its proinflammatory function in psoriasis [118,119,120]. 

The fact that periodontitis is reported to be twice as frequent and severe in rheumatoid arthritis patients compared to healthy controls indicates the correlated inflammatory processes in rheumatoid arthritis and periodontitis [121]. Accordingly, IL-17 levels were found to be increased in the gingival crevicular fluid of rheumatoid arthritis patients, further contributing to the inflammatory response in the gingival sulcus and the severity of periodontal inflammation [121,122]. Several links between rheumatoid arthritis and periodontitis have been suggested. One of the most striking ones is the citrullinated peptides production induced by *P. gingivalis*. Citrullinated peptides are considered to break tolerance and induce ACPA production in RA [123]. *P. gingivalis* is currently the only bacteria that is known to produce peptidyl arginine deiminase (PAD), an enzyme that leads to citrullination of the human and bacterial proteins [124]. In addition, the antibody titer against *P. gingivalis* was significantly increased in RA-patients, further supporting the role of this periodontal pathogen not only in periodontitis, but also in RA pathogenesis [125]. 

### 3.3. IL-17 Dependent Processes in Inflammatory Bowel Diseases and Association with Periodontitis

Inflammatory bowel diseases (IBD) are chronic inflammatory conditions of the gastrointestinal system and consist of ulcerative colitis (UC) and Crohn’s disease (CD). Ulcerative colitis is characterized by the chronic mucosal inflammation of the colon that manifests itself with abdominal pain, haematochezia, and diarrhoea [126,127]. In Crohn’s disease, however, any part of the gastrointestinal tract can be afflicted. This disease can typically be associated with extra-gastrointestinal symptoms such as anaemia, arthritis, skin rashes, oral lesions, and eye inflammations [128,129]. Although the etiology of IBDs remains largely unclear, a dysbiotic intestinal microbiome and risk factors, such as smoking and diet, were suggested to contribute to the disease onset via activation of inflammatory pathways that results in the disruption of the epithelial barrier integrity in genetically susceptible individuals [130]. The involvement of IL-23 and IL-17 in IBD is well documented; however, the different functions of IL-17 in IBD are still controversially discussed in the literature [131,132]. On the one hand, IL-17 deficient or anti-IL-17 treated mice exhibited severe epithelial damage in the colon, indicating a protective function of IL-17 [133]. This is further substantiated when inactivation of IL-17 resulted in a milder course of disease in an animal model of UC [134]. On the other hand, high IL-23 receptor and IL-17 mRNA expression levels were detected in intestinal mucosa samples of patients with active UC and CD [135,136]. Furthermore, many other studies reported increased levels of IL-17 in the intestinal mucosa and serum of active UC and CD patients [137,138]. 

Oral manifestations and implications of inflammatory bowel diseases are reported in a varying range from 0,5% to 37% among diseased individuals; they may appear as the first signs of the disease, especially in children, and include edema, mucogingivitis, oral ulcers, and hyperplastic lesions among others [139,140,141]. Involvement of upper regions of gastrointestinal tract and extra-gastrointestinal symptoms predict a more severe phenotype of the disease and may present with comorbidities due to the increased risk of systemic involvement [142]. Caries and periodontitis prevalence are reported to be often higher in individuals with CD and UC [143]. In a large nationwide cohort study, the prevalence of periodontitis was reported to be higher in patients with CD, with a hazard ratio of 1.36 (95% CI = 1.25–1.48) compared to the control group [144]. Similarly, a meta-analysis of cross-sectional studies, including a total of 1297 subjects, reported a significantly higher prevalence of periodontitis as well as a worse decayed-missing-filled-teeth index in patients with CD and UC compared to non-IBD individuals [145]. Interestingly, worse clinical periodontal parameters were observed among smokers with UC compared to smokers with CD [143]. Unfortunately, studies regarding the effect of periodontal inflammation on CD or UC currently remain deficient [146].

### 3.4. IL-17 Dependent Processes in Other Immune-Mediated Inflammatory Diseases and Association with Periodontitis

IL-17 also plays an important role in the pathogenesis of other IMIDs, such as Sjögren syndrome, systemic lupus erythematosus, and type 1 diabetes, among others. Sjögren syndrome is an autoimmune disease characterized by diffuse lymphocyte infiltration into exocrine glands that results primarily in xerostomia and ocular dryness, known as “sicca symptoms” [147]. Extra-glandular tissues and organs, such as skin, lungs, nervous system, kidneys, and the gastrointestinal tract are also affected by Sjögren syndrome in at least 30% of patients, classifying it as a systemic disease [148]. Sjögren syndrome can appear independently of other conditions as a primary disease (primary Sjögren syndrome) or manifest itself secondarily as a late complication with sicca symptoms (secondary Sjögren syndrome) in the presence of other systemic conditions, such as rheumatoid arthritis, systemic lupus erythematosus, or scleroderma [149,150]. In Sjögren syndrome affected tissues, several subsets of B and T cells can be identified, predominantly consisting of CD4+ T helper cell subtypes, such as Th1, Th17, and Tfh cells [151]. The interaction between epithelial cells, dendritic cells, and B and T cells results in an increased production of Tfh and Th17 cells, which intensifies the inflammation and increases autoantibody production [152]. Tfh cells govern the ectopic germinal center formation in salivary glands and potentiate the production of autoantibodies from B cells, whereas Th17 cells secrete IL-17 and IL-22 and stimulate inflammation [153,154]. Increased levels of IL-17 in plasma of patients with primary Sjögren syndrome, as well as an abundant presence of IL-1β, TGF-β, IL-6, and IL-23 in tissues affected by the disease, demonstrate the significance of Th17 cells and IL-17 in the disease pathogenesis [155,156,157]. Non-surgical periodontal therapy was demonstrated to improve the salivary flow rate and decrease the subjective disease activity index in primary Sjögren syndrome patients with periodontitis [158]. However, despite this finding and common IL-17-dependent pathways in periodontitis and Sjögren syndrome, a significant association between both diseases remains to be confirmed [159,160].

Systemic lupus erythematosus (SLE) is a connective-tissue disorder characterized by T cell abnormalities and production of a wide array of autoantibodies directed against double-stranded (ds) DNA. It affects multiple tissues and organs, often causing glomerulonephritis, arthritis, and blood cell abnormalities [161]. The interaction of Th17 cells, Tfh cells, extrafollicular T helper cells (eTfh, a Tfh cell analogue CD4^+^ subpopulation), Tregs, CD8^+^ cells, B cell subsets, and innate immune system cells results in a reduced IL-2- and an increased IL-17-production, as well as increased antibody-production from B cells [161,162,163]. Since IL-17, IL-6, and IL-33 were found to be significantly elevated in the saliva of SLE/periodontitis subjects compared to periodontitis-only subjects, this systemic imbalance of cytokines may have an impact on periodontal tissues [164]. However, the long-term corticosteroid therapy may also have resulted in the observed periodontal damage [164]. In a nation-wide retrospective population-based study, the history of periodontitis was associated with an increased risk of SLE; however, the common risk factors, such as smoking status, were not adjusted [165]. Conversely, periodontal treatment in SLE/periodontitis subjects was reported to significantly improve the responsiveness of SLE patients to the immunosuppressive therapy compared to the control group [166]. 

The autoimmune destruction of insulin-producing β cells in the Langerhans islets of pancreas results in type 1 diabetes, which is characterized by an insufficient insulin production leading to persistent or recurrent hyperglycemia [167]. Often diagnosed in childhood, type 1 diabetes causes lifelong dependence on insulin, as well as an increased risk of cardiovascular disease, neuropathy, nephropathy, and other autoimmune and inflammatory conditions such as rheumatoid arthritis [168]. CD4^+^ cells, especially Tregs, and CD8+ cells, autoantibody-producing B cells, and innate immune cells are involved in the disease pathogenesis [167]. IFNγ, produced by the infiltrated T cells, is a major cytokine involved in the destruction of the β cell islets; however, IL-17 was also demonstrated to play a role, when its neutralization prevented further disease development in 10-week old non-obese diabetic mice by reducing peri-islet T cell infiltration [169]. Also, increased secretion and expression of IL-17 and IL-22 were demonstrated to contribute to the disease development in type 1 diabetic children [170]. Impaired IL-2 functions that affect Treg functions lead to increased production of IL-17 and contributes to disease development in an animal model of type 1 diabetes [171]. Uncontrolled diabetes has been proven to be a predisposing risk factor for the onset and progression of periodontitis [172]. Conversely, the presence of periodontitis is suggested to affect the insulin metabolism by increasing the circulatory levels of proinflammatory cytokines [173]. Eventually, the treatment of periodontal disease has been shown to improve glycemic control, further substantiating the existing bidirectional relationship between diabetes and periodontal disease [174].

It is noteworthy to mention that the role of IL-17 has also been demonstrated in other IMIDs, such as ankylosing spondylitis, multiple sclerosis, Behcet disease, or scleroderma, in which aberrant immune and inflammatory responses also result in disease manifestation via similar mechanisms [175,176,177,178]. Unfortunately, conflicting data exists on the association of periodontitis and multiple sclerosis. In this regard, a large case control study found that no association between periodontitis and multiple sclerosis was seen between the groups after adjustment of covariates [179]. On the other hand, previous diagnosis of periodontitis was reported to be higher among female multiple sclerosis patients after adjustment of risk factors (odds ratio = 2.08; 95% CI, 1.49–2.95) [180]. In patients with scleroderma and periodontitis, a higher number of missing teeth and more clinical attachment loss were reported compared to patients with periodontitis only [181]. An association between Behcet disease severity and worse periodontal disease parameters (clinical attachment loss, bleeding on probing, and pocket probing depth) was also demonstrated in a cross-sectional study [182]. 

Based on the multiple interactions between genetic and environmental factors and aberrant immunological responses, multiple associations may exist between immune mediated inflammatory diseases and periodontitis. However, whether these association are causative and mutual needs to be further investigated. The findings of this research will expand the knowledge about the pathogenesis of IMIDs and periodontitis, and may help to improve the prevention, diagnosis, and the treatment of their systemic and oral complications.

## 4. Th17/IL-17 as Targets in the Management of IMIDs and Its Implications on Periodontal Inflammation

The treatment of immune-mediated inflammatory diseases is traditionally based on the use of glucocorticoids, non-steroidal anti-inflammatory drugs (NSAIDs), and disease-modifying antirheumatic drugs (DMARDs), such as methotrexate (MTX). They are proven to be effective against clinical signs and symptoms and are prescribed as the first-line therapy. However, intolerance, ineffectiveness, and severe adverse effects created the need for developing alternative therapies. Hence, in the early 1990s, monoclonal antibodies (mAbs) and fusion proteins, referred to as biologics or biological agents, were introduced. Biologics are a group of immunosuppressive drugs and are produced from a single clone of plasma B cells by modifying the Fc domain structures of its immunoglobulins. Initially, mAbs were only of murine origin and exhibited a reduced affinity due to the variability of the interaction of murine Fc with human Fc receptors, as well as caused the development of human anti-mouse antibodies [183,184,185]. To overcome these drawbacks, mAbs are nowadays bioengineered via recombinant techniques to produce chimeric, humanized, and fully human mAbs [186].

Biologics revolutionized the treatment of a wide range of immune-mediated inflammatory diseases, including but not limited to psoriasis, rheumatoid and psoriatic arthritis, ankylosing spondylitis, and inflammatory bowel diseases (Table 1). The first biologics that were introduced in the market were the TNF antagonists (Table 1). TNF antagonists act by inhibiting the binding of TNF to its receptor [186]. There are currently five TNF antagonists available, i.e., etanercept, infliximab, adalimumab, certolizumab, and golimumab. Besides others, TNF antagonists reduce IL-17 controlled inflammatory pathways (Figure 3a,b), and thus, slow down joint destruction in rheumatoid arthritis, reduce psoriatic lesions, as well as resolve chronic intestinal inflammation and ulcerations in IBD [183,187,188]. Etanercept, a TNF receptor-2 and IgG1-Fc fusion protein, reduces infiltration of polymorphonuclear cells into gingival tissues and improves periodontitis parameters in a murine model of experimental periodontitis [189]. In contrast to these anti-inflammatory functions, TNF antagonists interestingly can also cause the appearance of psoriasis-like lesions in 5–10% of treated patients, named as ‘paradoxical psoriasis’ [129,190]. Paradoxical psoriasis is suggested to be a side effect of all TNF antagonists, irrespective of the type and dosage, and usually disappears upon discontinuation [190,191]. In addition to paradoxical psoriasis, TNF inhibition was reported to increase susceptibility to bacterial infections [192]. The pharmacokinetic and pharmacodynamic properties differ among TNF antagonists as a result of their different molecular structures and mode of administration. Therefore, their effects show a considerable variability among individuals and diseases. Several reports indicate that in case of an ineffective response or intolerance to a specific TNF antagonist, the treatment can be continued with another TNF antagonist or replaced by a different biological drug, such as an IL-17 inhibitor [183,193]. It is noteworthy to mention that periodontitis is associated with an increased risk of etanercept discontinuation with an hazard ratio of 1.27 (95% CI, 1.01–1.60) in anti-TNF-naïve rheumatoid arthritis patients if they have been diagnosed with periodontitis within 5 years prior to or during etanercept treatment [194]. 

**Figure 4:**
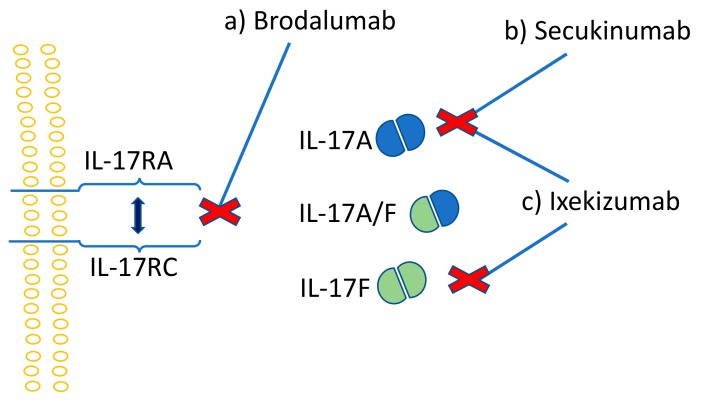
IL-17 cytokine inhibitors; (**a**) brodalumab, with affinity to IL-17RA/RC receptor complex, (**b**) secukinumab, with affinity to IL-17A, and (**c**) ixekizumab, with affinity to both IL-17A and IL-17F, interrupt the intracellular signaling by inhibiting the interaction between IL-17 and its receptor.

Due to the synergistic effects of IL-17 and TNF in inflammation, diseases that are treated with TNF antagonists can also be managed with mAbs that target IL-17 or the IL-23/IL-17 axis [195]. Moreover, the inhibition of both TNF and IL-17 was shown to be more effective compared to TNF or IL-17 inhibition alone [196]. The first introduced anti-IL-17 drug was secukinumab, a human IgG1k monoclonal antibody, which acts by binding directly to IL-17A and inhibits its action (Figure 4b). Ixekizumab is a slightly different antibody that is developed from IgG4k, with affinity only to the IL-17A homodimer and IL-17A/F heterodimer of the IL-17 family (Figure 4c). Similarly to secukinumab, ixekizumab inhibits the IL-17 action by binding directly to this cytokine. Brodalumab, a human IgG2k mAb and the first anti-IL-17 receptor agent, inhibits the action of IL-17 by binding to the IL-17 receptor (Figure 4a) [197]. Phase II and III clinical studies proved IL-17 inhibitors as safe and efficient for the treatment of psoriasis, rheumatoid arthritis, and inflammatory bowel disease [198,199,200,201]. In psoriasis, however, the efficacy of ixekizumab was shown to be significantly superior when compared to etanercept and ustenikumab (IL-12/23 mAb) [202,203].

Comparative reviews on anti-IL-17 drugs reported that all three drugs were well-tolerated in patients with moderate-to-severe psoriasis; however, adverse effects, such as respiratory tract infections, were reported more frequently for ixekizumab, and it was withdrawn in some cases due to its toxicity [204,205]. A meta-analysis on efficacy of anti-IL-17 drugs on rheumatoid arthritis showed that secukinumab and ixekizumab were more effective compared to placebo; however, an increased risk of infection in the test group has also been reported [206]. Low levels of IL-17 in the synovium, heterogeneity in expression patterns of IL-17 and its receptors were shown to reduce anti-IL-17 drug effect in animal model studies, which could be the explanation to the insufficient efficacy in some patients [207]. 

Since the maintenance and expansion of Th17 cells are IL-23-dependent, the inhibition of IL-23 can also reduce IL-17 production and accomplish a clinical improvement. Ustekinumab is a human IgG1k monoclonal antibody that binds to the p-40 subunits of both IL-23 and IL-12 and interferes with the binding to their receptors. Long-term, multicenter, placebo-controlled phase III studies demonstrate the safety, efficacy, and superiority of ustekinumab to etanercept in patients with moderate to severe psoriasis [208,209,210]. However, an increased risk for neoplasia during IL-12/23 inhibitor use was reported in animal studies, which was attributed to IL-12 involvement in tumor surveillance [211,212]. Ustenikumab has also been demonstrated to reduce inflammatory responses in a patient with leukocyte adhesion deficiency type 1 (LAD1) periodontitis without serious adverse effects already after 3 weeks of treatment [213]. In addition to IL-12/IL-23 inhibitors, drugs such as guselkumab and tildrakizumab were developed to target the unique p19 subunit of IL-23 (Figure 1d,e). As expected, Th17-cell infiltration was decreased in psoriasis patients while anti-inflammatory IL-10 expression from Th1-cells was increased following the administration of IL-23 inhibitors [214,215]. 

As mentioned before, an increased risk of infections, especially by *Candida* spp., is often reported among patients that use these biologics over a prolonged time. Nevertheless, it is noteworthy to mention that overexpression of inflammatory cytokines may also increase infection risk, as periodontal pathogens were suggested to thrive in a highly proinflammatory cytokine environment [59]. 

## 5. Conclusions

IL-17 plays an important role in inflammatory events that lead to the manifestation of psoriasis, rheumatoid arthritis, inflammatory bowel disease, and periodontitis. Although much remains to be clarified regarding its protective and pathologic functions, the current knowledge suggests its role as a potent proinflammatory mediator and bridge between innate and adaptive immune responses. Comorbid periodontitis is often observed in patients diagnosed with an immune-mediated inflammatory disease. Although a bidirectional causal relationship is yet to be confirmed, regular screenings, preventive measures, and early treatment could reduce the burden of periodontitis in these patients, and vice versa. Many clinical randomized controlled trials prove the efficacy of cytokine inhibitors that manipulate IL-17 and related pathways in the management of psoriasis, rheumatoid arthritis, and inflammatory bowel disease. Unfortunately, reports regarding the therapeutic effects of cytokine inhibitors on gingival, periodontal, and oral mucocutaneous diseases are scant, which could be due to their restricted indication for severe systemic conditions, high costs, and adverse effects. However, further clinical research on the effects of biologics on gingival, periodontal, and oral tissues is needed to further elucidate the role of Th17-cells and the IL-23/IL-17 axis in the pathogenesis of periodontitis and its potential association with IMIDs. 

## Figures and Tables

**Table 1:** Target cytokines involved in IL-17 pathways and approved inhibitors in the management of immune-mediated inflammatory diseases.

Targeted Cytokine	Drug	Indicated Conditions
TNF	Etanercept	Psoriasis, RA, PsA
	Infliximab	Psoriasis, RA, PsA, ankylosing spondylitis (AS), Crohn’s disease (CD), ulcerative colitis
	Adalimumab	Psoriasis, RA, PsA, AS, CD, uveitis, hidradenitis suppurativa
	Certolizumab	Psoriasis, RA, PsA, AS, CD,
	Golimumab	RA, PsA, CD, ulcerative colitis, AS
IL-1	Anakinra	RA
IL-6	Tocilizumab	RA
IL-12/23	Ustenikumab	Psoriasis, PsA, CD
IL-23	Guselkumab	Psoriasis
	Tildrakizumab	Psoriasis
IL-17	Secukinumab (IL-17A)	Psoriasis, RA, PsA, CD, asthma, AS, uveitis, multiple sclerosis
	Ixekizumab (IL-17A)	Psoriasis, RA
	Brodalumab (IL-17RA)	Psoriasis, PsA, RA, Crohn’s disease, asthma
